# Solution NMR Studies on the Orientation of Membrane-Bound Peptides and Proteins by Paramagnetic Probes

**DOI:** 10.3390/molecules18077407

**Published:** 2013-07-25

**Authors:** Evelyne Schrank, Gabriel E. Wagner, Klaus Zangger

**Affiliations:** Institute of Chemistry/ Organic and Bioorganic Chemistry, University of Graz, Heinrichstrasse 28, A-8010 Graz, Austria

**Keywords:** NMR spectroscopy, membrane-bound peptides and proteins, paramagnetic relaxation, micelle, dodecylphosphocholine

## Abstract

Many peptides and proteins are attached to or immersed in a biological membrane. In order to understand their function not only the structure but also their topology in the membrane is important. Solution NMR spectroscopy is one of the most often used approaches to determine the orientation and localization of membrane-bound peptides and proteins. Here we give an application-oriented overview on the use of paramagnetic probes for the investigation of membrane-bound peptides and proteins. The examples discussed range from the large pool of antimicrobial peptides, bacterial toxins, cell penetrating peptides to domains of larger proteins or the calcium regulating protein phospholamban. Topological information is obtained in all these examples by the use of either attached or freely mobile paramagnetic tags. For some examples information obtained from the paramagnetic probes was included in the structure determination.

## 1. Introduction

Membrane-bound proteins and peptides constitute ~30% of all naturally occurring proteins and peptides [1]. Some of them, in particular G protein coupled receptors, are the target of almost half of all pharmaceutical drugs [2]. However, a very low percentage of structurally characterized proteins are membrane-bound [3], which results from difficulties in their preparation, crystallization and NMR spectroscopic characterization. Nevertheless, in recent years the number of membrane-bound protein structures determined by solution NMR spectroscopy has significantly increased. One particular promising new approach is the combination of cell-free protein synthesis with combinatorial dual labeling and paramagnetic relaxation enhancement restraints [4]. In addition, atomic resolution structures of small membrane-bound peptides can be obtained on a routine basis by solution NMR spectroscopy. Besides the structure, the localization and orientation in the membrane is often even more important to get insight into the biological function(s) of these hydrophobic peptides and proteins [5]. A plethora of biophysical techniques is available to determine the orientation of proteins and peptides in a membrane or membrane-mimetic, with magnetic resonance methods being arguably the most commonly used approach. Solution NMR spectroscopy using paramagnetic probes offers a particularly convenient technique to investigate the topology of membrane associated proteins and peptides. It does not require a chemical modification of the peptide or protein to be studied and appropriate hardware is available at basically all research institutions. However, one of the major disadvantages of solution NMR is its size limitation, which makes obtaining atomic resolution structural information of large molecules or molecular assemblies very difficult. Although this size limit could be significantly extended in the last dozens of years by techniques like transverse relaxation optimized spectroscopy (TROSY) [6] or selective, relaxation-optimized isotopic labeling [7], atomic resolution structural studies on systems above ~ 40 kDa are still rather challenging. For this reason membrane-bound peptides and proteins have to be studied using very small membrane-mimetics. By far the majority of solution NMR studies on membrane-bound molecules have been carried out using micelles [8,9,10], typically sodium dodecylsulfate (SDS), dodecylphosphocholine (DPC) or dihexanoylphosphatidylcholine (DHPC) due to their availability in deuterated form. For some NMR studies bicelles or small vesicles were used [11,12] and very recently phospholipid bilayer nanodiscs were introduced for solution NMR studies of membrane-bound proteins [13,14]. They consist of small (diameter ~7–10 nm) assemblies of phospholipids, surrounded by apolipoprotein A-1 or proteins derived from it.

Addition of a paramagnetic probe, either to the solvent or the membrane-mimetic results in relaxation enhancements [15], which depend on the distance between the paramagnetic center and the nucleus for which the relaxation enhancement is measured. In many studies paramagnetically tagged lipids were used, which yield higher PREs for nuclei, which are farther inserted in the membrane-mimetic [16]. As an alternative, inert paramagnetic compounds in the solvent show which signals are closer to the surface of the membrane [17]. Here we review applications on the use of paramagnetic probes to obtain information about peptide and protein structure and/or orientation in membrane-mimetics by solution NMR spectroscopy. Considering the huge number of such reports in the literature we have to restrict this overview to some, mainly recent, examples from different classes of peptides and proteins, many of which also report on new methodological advances in the field.

## 2. Paramagnetic Agents

### 2.1. Paramagnetically Tagged Lipids

**Figure 1:**
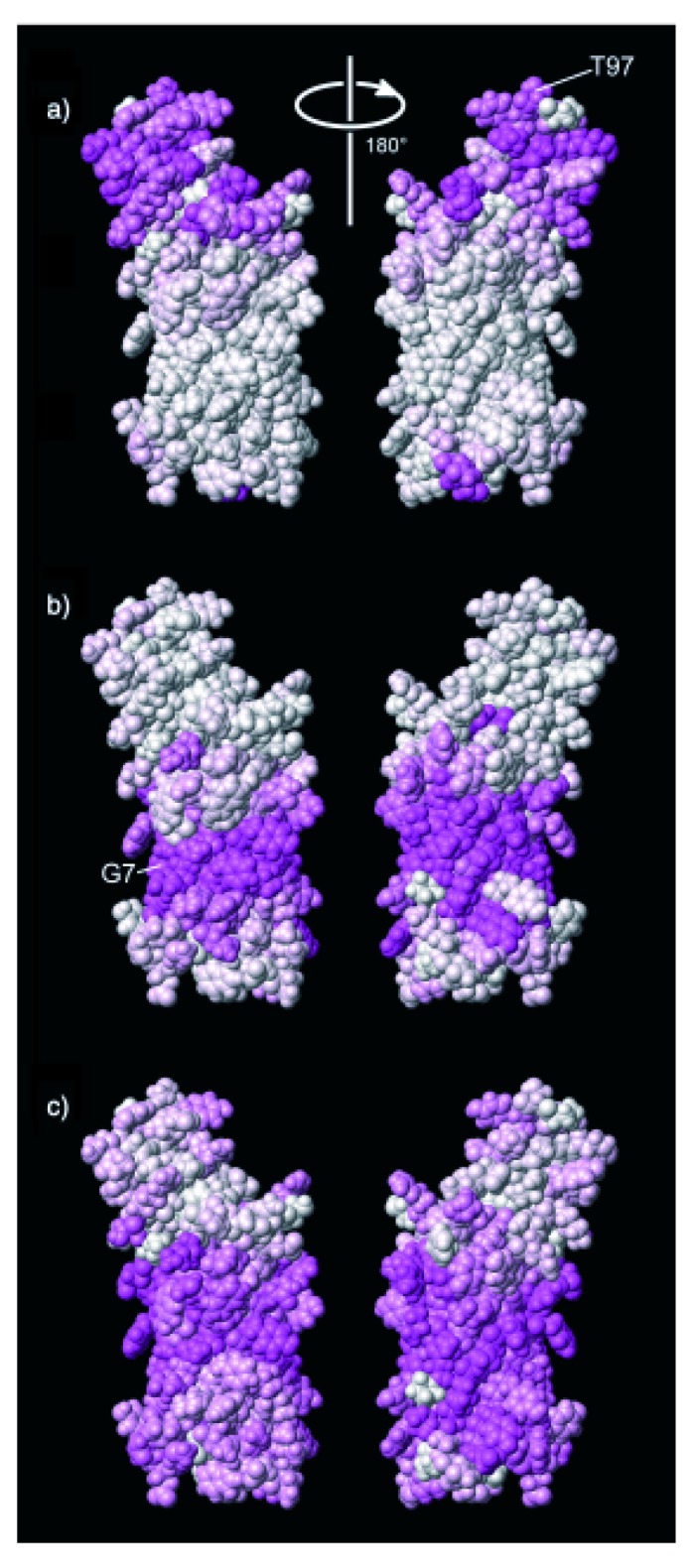
Paramagnetic relaxation enhancements of (**a**) Gd(DOTA), (**b**) 16-DSA and (**c**) 5-DSA on the membrane-bound protein OmpX. The darkness of the color indicates the size of the PREs with dark purple corresponding to highest PREs. Reproduced with permission from [19].

The earliest study on the topology of a membrane-bound peptide in a micelle was the binding of the 29 residue peptide glucagon to DPC micelles by the group of Wüthrich [16]. The spin labels 5-doxyl-stearate (5-DSA), 12-doxylstearate (12-DSA) and 16-doxylstearate (16-DSA) were added at a concentration of roughly one label per micelle. They provide a paramagnetic center at different locations in the DPC micelles, where the nitroxyl group is immersed deepest by using the 16-doxylstearate and closest to the surface by 5-doxylstearate. These paramagnetic positions could also be seen on the ^13^C signals of DPC. For glucagon, locations close to the surface were found for some nuclei in residues 1, 12, 17, 18 and 29 indicating that the whole peptide is bound basically parallel to the surface with some residues being inserted deeper in the micelle than others. After this seminal report, the use of doxylstearate became one of the most frequently used approaches to investigate the topology of membrane-bound peptides and proteins by solution NMR spectroscopy. Another nice example from the Wüthrich group was the combined use of doxylstearate and the gadolinium complex Gd(DOTA) (Gd^3+^ complexed with 1,4,7,10-tetraazocyclododecane-N,N',N'',N'''-tetraacetic acid) for determining the orientation of the *E. coli* outer-membrane protein X (OmpX) in DHPC micelles [18,19]. Differences in line-broadening in ^1^H-^15^N HSQCs provided information about which parts of this ~16 kDa membrane protein are located in the micelle (see Figure 1). The results were in very good agreement with the topology obtained by NOEs between the protein and the micelle forming lipids. 

One very recent example of the use of paramagnetically tagged lipids is its application for reducing longitudinal relaxation times and thereby speeding up NMR experiments [20,21]. Copper-chelated 1,2-ditetradecanoyl-sn-glycero-3-phosphoethanolamine (DMPE) can be inserted in the membrane mimetic and leads to enhanced T_1_ relaxation while T_2_ relaxation is less influenced.

### 2.2. A Paramagnetic Insertion Gradient by Dissolved Oxygen

At high partial pressures (20–100 bar) oxygen displays a concentration gradient in micelles with higher concentrations in the hydrophobic interior [22,23,24]. This paramagnetic contrast effect is distributed Gaussian-shaped and causes higher paramagnetic relaxation enhancements (PREs) and induced chemical shift changes the closer a nucleus is to the micelle’s center. This approach has been developed in the group of Scott Prosser and was first applied to ^19^F-NMR of small fluorinated membrane-bound molecules [24] and the integral membrane protein diacylglycerol kinase (DAGK) [25]. Single cysteine mutants of this 39 kDa homotrimeric protein were prepared and allowed to react with 3-bromo-1,1,1-trifluoropropanone to introduce fluorinated groups into the transmembrane helix 1. Fluorine-19 chemical shifts are very sensitive to any changes in their environment. The effect of oxygen on ^19^F also leads to different spin-lattice relaxation times and line widths. Changes of these parameters by increasing the oxygen pressure as a function of residue number yield an oscillating function with a periodicity of ~ 3.6 residues from which the tilt angles of membrane-bound helices can be obtained by least-square fitting. This method was applied to confirm the previously proposed 25° tilt angle of the membrane-bound β-barrel protein PagP in DPC micelles [26]. For this study amide PREs were obtained by measuring the relaxation behavior of longitudinal 2H_Z_N_Z_ two-spin order, rather than proton magnetization. This is necessary in order to avoid exchange with water to influence the experimental PREs. 

### 2.3. Inert Gadolinium-Based Solvent PREs and Paramagnetic Relaxation Waves

While interactions between paramagnetic metal ions like Mn^2+^ with negatively charged groups in micelles and membrane-bound peptides and proteins cannot be excluded [27,28], gadolinium-diethylenetriamine pentaacetic acid-bismethylamide [Gd(DTPA-BMA)], the active component of the MRI contrast agent Omniscan^©^ has been shown to be inert towards proteins [29] and also DPC micelles [30,31]. Addition of this paramagnetic probe leads to solvent PREs, which simply depend on the insertion depth into the membrane-mimetic [29,32]. While these solvent PREs could be obtained on any NMR active nucleus, the effect is stronger for ones with high gyromagnetic ratio, like ^1^H and ^19^F [32,33,34]. The first rather qualitative application of Gd(DTPA-BMA) to membrane-bound peptides was its use to determine the orientation of the α-helical antimicrobial peptides bombinin H2 and H4 [35]. Its influence on the relaxation was obtained by measuring peak intensities in the absence and presence of Omniscan^©^. Since these values were similar for the central part of the peptides, as well as the termini, an orientation parallel to the surface was inferred. To obtain the exact orientation of membrane-bound peptides and proteins by Gd(DTPA-BMA) a more quantitative evaluation of its paramagnetic relaxation enhancements is necessary. 

In order to define the orientation of an α-helix in a membrane or membrane-mimetic environment two parameters are necessary: the tilt angle τ, which defines the angle between the helix axis and the membrane surface normal and the azimuth angle ρ (also called rotation angle), which describes the rotation of the helix (*i.e.*, which side-chains point towards the interior). Using these variables the immersion depth d of a particular backbone nucleus of an α-helix is given by:

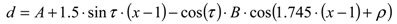
(1)
where the variable x is the residue number and A is the immersion depth of the first residue. The second term 1.5*sin(τ)*(x−1) describes the increasing membrane insertion by going along the peptide chain when the tilt angle is larger than zero. The factor of 1.5 is the helical pitch (in Å) per residue. The third term in Equation (1) accounts for the oscillating helical behavior and is given by cos(1.745x+ρ), where 1.745 × is equal to 2πx/3.6 and results from the periodicity of 3.6 residues per turn. B is the radius of the helix measured at the site of the nuclei under study. For a typical α-helical geometry this radius is 3.25 Å when measured at the site of Hα protons and 1.95 Å for NHs. The paramagnetic relaxation enhancement (PRE) exerted by a single paramagnetic center on a nucleus at a distance of r is given by a k/r^6^ dependence. Since we are interested only in the distance dependence, all other contributing factors are combined in the constant k. Addition of a paramagnetic agent to the solvent makes the whole environment of the membrane-mimetic paramagnetic. Therefore, the contributions of all paramagnetic centers need to be integrated, which results in a 1/d^3^ dependence of PRE on immersion depth d. The PRE as a function of helix positioning parameters is then given by:

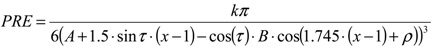
(2)

**Figure 2:**
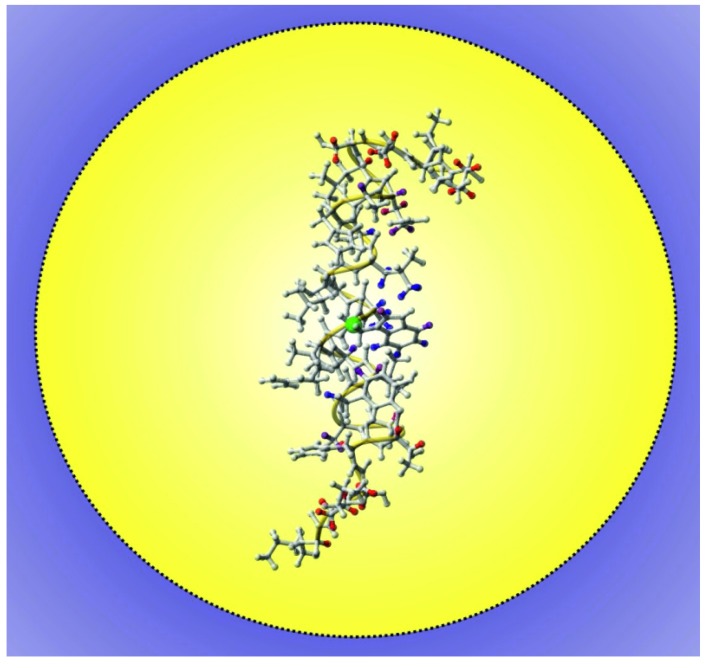
Localization and orientation of the transmembrane helix 7 (TM7) of yeast V-ATPase in DPC micelles as obtained by PREs induced by Gd(DTPA-BMA). The PREs are indicated by the color code on the hydrogen atoms for which they were determined with red indicating high PREs and therefore close proximity to the surface and blue is used for low PREs and localization deep in the micelle.

Equation (2) can be used to fit the measured relaxation enhancements as a function of residue number to obtain the exact positioning of a helical peptide in a membrane. For micelles the tilt angle is defined as the angle between the helix axis and a line connecting the micelle center with the helix center. If accurate relaxation times are used, the orientation of a structurally characterized peptide in a micelle can be obtained by least-square fitting. In the case of α-helical peptides the resulting paramagnetic relaxation wave provides the tilt and rotation angle in the micelle [17], even if no atomic resolution structure is available. This approach has been used, for example, to obtain the orientation of the designed antimicrobial peptide CM15 [17] and the transmembrane helix 7 of yeast V-ATPase (TM7) in DPC micelles [31]. CM15, which was completely assigned NMR spectroscopically [17,36], forms an amphipathic helix bound parallel to the surface, yields a paramagnetic relaxation wave with a periodicity of 3.6 residues corresponding to the number of residues per turn of an α-helix. In contrast, TM7 shows a steady decline of solvent PREs from each terminus of the helix towards the center of the peptide, which is observed for a trans-membrane (trans-micellar) orientation (see Figure 2).

Orientations parallel to the surface were also found for the antimicrobial peptide maximin H6 in both SDS and DPC micelles [37]. This amphipathic α-helical peptide showed generally higher solvent PREs in SDS compared to DPC micelles and ^15^N relaxation times indicated that this peptide is relatively rigid in SDS and even more in DPC micelles. While Gd(DTPA-BMA) can be used quantitatively to determine the exact positioning of an immersed peptide in a micelle, it can be also used to yield qualitative information on the membrane attachment for peptides or proteins, which are only partially inserted into a micelle. An example is the 28-residue hormone peptide ghrelin, which binds to DPC micelles via insertion of its octanoyl group on Ser-3 and the aromatic ring of Phe-4 [38]. Some, especially larger membrane-bound peptides and proteins possess both membrane-immersed and soluble parts. An example is the bacterial toxin Fst, whose ~27 N-terminal residues form a transmembrane-spanning helix and the last C-terminal residues are unstructured in solution [39]. The orientation in the membrane was obtained by limited solvent PREs and corroborated by molecular modeling.

## 3. Using PREs for the Structure Calculation of Membrane-Bound Peptides and Proteins

For the majority of reports paramagnetic relaxation enhancements were used to determine the orientation and localization of membrane-bound peptides and proteins. However, they can also be used for the actual structure elucidation. Wimmer *et al.* have used relaxation enhancements induced by Gd(DTPA-BMA) and calibrated them to distances from the micelles’ surface by comparison of the experimental PREs of DPC with the average position of the corresponding nuclei in modeled micelles [40]. The positioning in the micelle as well as the refined NMR structures of the antimicrobial peptides novicidin and novispirin were obtained by this approach. Both peptides show a very similar orientation of α-helices bound close to the surface of the micelles with paramagnetic relaxation waves confirming this topology. 

PREs from spin labels on a membrane protein can also be used as additional restraints for protein structure calculation, just as it is done routinely for soluble proteins. For example, an 80-residue fragment of the G protein coupled receptor Ste2p from *Saccharomyces cerevisiae*, containing two transmembrane helices was structurally characterized in TFE:water solution [41]. A cysteine residue was introduced and PREs measured after a 1-oxyl-2,2,5,5,-tetramethyl-D3-pyrroline-3-methyl methanethiosulfonate (MTSL) nitroxide spin label was attached to ^15^N, ^13^C, ^2^H (methyl-^1^H-ILV) labeled protein to obtain interhelical distance information.

A series of cysteine mutants of the 127 residue membrane protein Rv1761c from *Mycobacterium tuberculosis* were prepared to determine its structure in DPC micelles [42]. Again MTSL groups were attached and PREs obtained by monitoring intensity changes in ^1^H-^15^N HSQC spectra. To avoid significant inaccuracies in PREs to distance translations the spin labels were introduced at more rigid parts of the protein so that one overall rotational correlation time can be used to describe the PRE *versus* 1/r^6^ dependence. The structure of this protein is characterized by one kinked transmembrane helix and an extra membrane domain consisting of four helices, which are in contact with the membrane and not freely mobile outside the micelle. 

**Figure 3:**
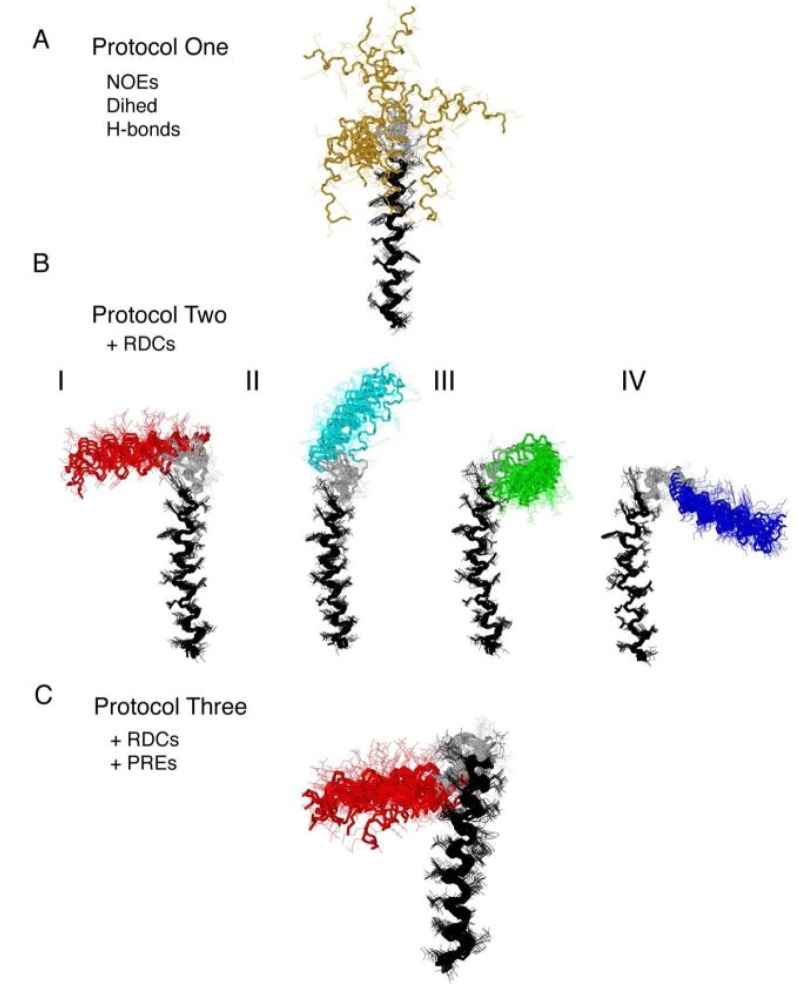
Structures of monomeric phospholamban obtained using (**a**) NOEs, dihedral angle restraints and hydrogen bonds, (**b**) all of the above plus residual dipolar couplings and (**c**) all of the above and PREs. Reproduced with permission from [43].

Paramagnetic relaxation enhancements from an attached spin label were also used by Veglia *et al.* to resolve the structural heterogeneity of the membrane protein phospholamban in DPC micelles [43]. The relative orientation of the two helical domains of this 52-residue protein in detergent micelles could not be obtained by NOEs exclusively. Including residual dipolar couplings yielded four possible orientations, which could be reduced to only one by addition of PREs induced by the spin label MTSL attached to residues 7, 9 and 24, which were mutated to cysteines (see Figure 3). Upon incorporation of a PRE energy term in the force field used for the structure calculation only the orientation, where the hydrophobic part of the N-terminal helix faces the other transmembrane helix shows a remarkably lower energy.

## 4. Applications

### 4.1. Phospholamban

Phospholamban is a 52-residue membrane-bound protein, which regulates Ca^2+^ levels in the cardiac sarcoplasmic reticulum by binding to sarcoplasmic reticulum calcium ATPase (SERCA) [44]. In biological membranes it occurs mainly as a homopentamer, in equilibrium with a small amount of monomer [45]. The monomer is the biologically active form that binds to SERCA. A zero cysteine mutant of phospholamban prevents the formation of the pentamer [46]. In both forms the protein consists of two α-helices connected by a turn and the NMR structures of both the monomer and pentamer in membrane-mimetics were determined by a combination of solution and solid-state NMR techniques [47,48,49]. The two helices are arranged in an L-shaped conformation. The orientation of monomeric phospholamban in DPC micelles was obtained using paramagnetic Mn^2+^ ions and 5- and 16-doxylstearic acids (DSA) [49]. Based on intensity changes of NMR signals in the presence of these paramagnetic agents it was concluded that both termini and the hinge region are located close to the micelle surface. The N-terminal helix is oriented basically parallel to the surface and the more hydrophobic C-terminal helix sticks through the micelle center. The structure of monomeric phospholamban in membrane-mimetics was later refined using distance, torsion angle and orientational restraints from both solution and solid-state NMR experiments and the exact orientation in DPC micelles has recently been determined by PREs of dissolved oxygen [50]. Using this approach the N-terminal amphipathic helix is oriented with a tilt angle of 87 ± 1° and the C-terminal with a tilt angle of 25 ± 4° relative to the micelle’s normal. The structure and orientation in the membrane of the pentameric form of phospholamban has been established using again a combined solution and solid-state NMR approach. The individual units of the pentamer again form an L-shaped conformation, which is arranged in a pinwheel topology [43], where the N-terminal (cytoplasmic) helix is oriented parallel to the surface of the membrane. For the pentamer orientation gadopentetate dimeglumine and 5- and 16-doxyl stearic acids were used. 

### 4.2. The Heat Shock Protein Hsp12

Hsp12 is a small (109 residues) heat shock protein in yeast. It is found both in the cytosol and the plasma membrane [51]. It is structurally completely different from all previously known heat shock proteins, because it is intrinsically unfolded but becomes structured upon interaction with membranes [52,53]. Hsp12 interacts preferentially with negatively charged membrane-mimetics. The formation of an α-helix was observed in zwitterionic DPC micelles, whereas it forms four helices in negatively charged SDS micelles [52]. Due to the absence of long-range structural restraints between the individual helices they seem to be connected by flexible loop regions. The orientation of the helices in SDS micelles was obtained by the groups of Buchner and Kessler using quantitative solvent PREs induced by Gd(DTPB-BMA) [53]. Helices 1 and 3 showed relatively high PREs of ~0.7 s^−1^mM^−1^, which is indicative of an orientation basically on the surface of the micelles, while the value of ~0.3 s^−1^mM^−1^ found for helices 2 and 4 indicate its shallow immersion into this membrane-mimetic. Paramagnetic relaxation waves showed the amphipathic orientation of all helices. Qualitatively the same results were obtained in the group of Markley [52], who used also Gd(DTPA-BMA) as well as 5- and 16-doxyl stearic acid to localize Hsp12 in SDS micelles. Peak intensity changes in HSQC spectra showed that all four helices are localized on the surface of the micelles. Hsp12 obviously stabilizes biological membranes by modulation of their fluidity. It remains to be seen if similar heat shock proteins will also be found in higher eukaryotes. 

### 4.3. Antimicrobial Peptides

Antimicrobial peptides (AMPs) can be found in almost all higher organisms [54,55]. They are typically short (8 to 50 amino acids), cationic peptides with a high percentage of hydrophobic residues and usually forming amphipathic structures when bound to a membrane [55,56,57]. In aqueous solutions AMPs are usually intrinsically unfolded, but upon membrane-binding they become structured and can be divided into one of the four structural groups, which are [58]: alpha helical peptides (e.g., maximin), extended structures (e.g., indolicidin), disulfide-stabilized beta-sheets (e.g., arenicin) and loop peptides (e.g., bactenecin). AMPs, as part of the innate immune system, act as a first unspecific response to pathogens, either by the permeabilization of their cell wall [54], interaction with the pathogen metabolism [59] or the modulation of the immune system [60]. Due to increasing problems with microbial antibiotic resistances as well as their broad spectrum of action against bacteria, fungi and other micro-organisms there is a high pharmaceutical interest in AMPs [58,61,62]. However, despite tremendous efforts to obtain rationally designed AMPs with well-specified properties, only a few of them have proven to be clinical efficient so far [58]. Therefore, further insights into the mechanisms of membrane association, permeabilization and the factors that influence antimicrobial activity are required.

**Figure 4:**
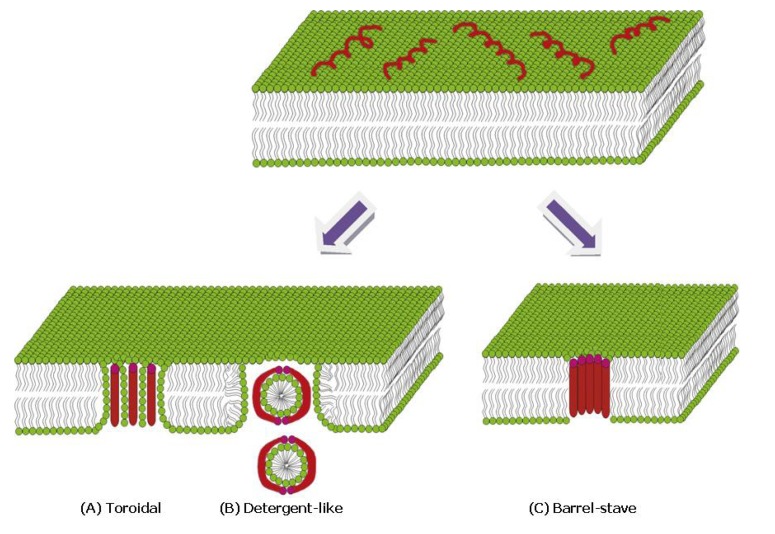
Proposed modes for membrane permeabilization by AMPs: (**A**) Formation of a toroidal pore, where the peptide as well as the detergent head groups face the interior. (**B**) Solubilization of membrane patches by a detergent-like mechanism. (**C**) The AMPs form an ion-channel-like pore, where only the hydrophilic residues of the peptide face the solvent. Adopted with permission from [62].

The mechanism of membrane disruption by AMPs is not yet fully understood [62], although previous studies led to the proposal of the following models: In the toroidal pore model, AMP binding leads to a positive curvature of the membrane, resulting in a pore where the peptides as well as the lipid head groups face the interior of the pore. In the barrel stave model the oligomerization of the peptides in a transmembrane spanning orientation leads to ion-channel-like structures, where only the hydrophilic residues of the peptide cover the pore. Finally, also a detergent-like mechanism has been proposed, where AMPs accumulate and solubilize patches of the membrane by stabilizing toroidal aggregates at a certain peptide concentration (see Figure 4) [62].

Still, all these models cannot provide an explanation for the membrane specificity of the peptides. One striking difference to the anionic outer membrane of bacteria is that eukaryotic ones mainly consist of neutral and zwitterionic lipids and as a result do not have a net charge, which diminishes the interaction with the cationic AMPs [63]. Nevertheless, side effects on eukaryotic cells cannot be ruled out as, for example, hemolytic activity was shown for a number of AMPs like melittin [64] and maximin H6 [37]. For a better understanding of AMPs not only the 3D atomic resolution structure but also the dynamics, immersion depth and the orientation of the peptides relative to the membrane are of interest. Besides electron paramagnetic resonance (EPR) [65,66] and solid state NMR spectroscopy [67,68], especially solution NMR spectroscopy here has proven to be an incredibly versatile tool in particular in combination with paramagnetic agents. The following examples will give a brief overview of the application of these probes and the conclusions drawn by the studies.

#### 4.3.1. α-Helical Peptides

The majority of AMPs form α-helical structures in membranes and their topologies, including the oligomerization state, have been extensively studied by paramagnetic relaxation experiments [69,70,71].

##### 4.3.1.1. Temporins

An interesting example is the family of temporins obtained from frog skin, which includes the shortest (8 to 13 amino acids) AMPs currently known [56,72]. Temporin-SHa (SHa) and temporin-SHc (SHc) share similar sequences, but differ in their single basic residue (lysine instead of histidine), which might be an explanation for SHa’s broader antimicrobial range. Furthermore, temporin-SHb (SHb), which lacks a basic residue, shows no antimicrobial activity at all [73]. The solution structures of temporins in negatively charged SDS micelles show amphiphilic α- helical conformations [73]. In order to address the question whether the localization within the micelle could explain their different antimicrobial activities Mn^2+^ and 5-doxyl stearic acid were used. Significant signal broadening can be observed all over the peptides with both paramagnetic agents, indicating localization close to the micelle’s surface. Furthermore, wavelike patterns of the PRE confirm the amphiphilic nature of the AMPs and an orientation parallel to the membrane surface. However, in contrast to SHa and SHc, in SHb the least Mn^2+^-affected part shifts closer to the N-terminus. Another example is temporin-SHf (SHf) with a length of only eight amino acids it is the shortest known linear AMP [56]. It is very hydrophobic compared to other AMPs and contains four phenylalanines. SHf possesses antimicrobial activity against many bacteria and yeast, and yet shows no hemotoxicity. NMR studies revealed an α-helical conformation and a micellar orientation similar to the above mentioned temporins. [56] The length of the peptide permits a transmembrane orientation therefore the authors propose a detergent-like mode of action supported additionally by DSC (differential scanning calorimetry) measurements.

##### 4.3.1.2. Melittin

As part of the honey bee’s venom, melittin causes cytolytic reactions besides its favorable antimicrobial activity. Saravanan *et al.* investigated the influence of hinge-region deletions and D-amino acid substitution on its antimicrobial properties in order to avoid hemotoxic side effects [74]. The first construct, Mel-H, lacked the hinge region and the two last C-terminal amino acids and showed decreased hemotoxicity with unchanged antimicrobial activity. These characteristics can be amplified further by the additional replacement of four residues with their D-enantiomers (Mel-dH). The structures of both peptides in DPC micelles show a helical structure for Mel-H and a compact, only partially helical one for Mel-dH. This indicates that the kink in the full length structure of melittin is not necessarily an artifact of the micelle’s curvature and that its hinge region plays a crucial role in hemolytic activity. Besides the structural characterization, the immersion depth was assayed by 5- and 16-DSA [74]. The results show that Mel-H is mainly affected by 16-DSA, indicating a localization close to the micelle’s interior, and Mel-dH by both probes. The observed PREs can be interpreted as being a result of the higher degree of flexibility of Mel-dH. This is in agreement with the much faster amid proton deuterium exchange rates of Mel-dH compared to Mel-H. Obviously, Mel-dH’s larger dynamics and its proximity to the membrane surface are the reasons for the reduction of its hemotoxicity.

##### 4.3.1.3. Magainin

MSI-78 and MSI-594, two synthetic AMPs based on magainin, were studied regarding the relationship between dimerization and antimicrobial activity [68]. Oligomerization seems to be a vital step for the activity of magainins, therefore MSI-594, lacking the phenylalanine zipper dimerization motif, is an excellent model system to address this issue. NOESY spectra confirmed the mainly α-helical conformation of both peptides in DPC micelles. However, in the case of MSI-78 additional NOESY peaks can be observed, that originate from antiparallel MSI-78 dimer formation. This is consistent with solid-state NMR measurements of MSI-78 bound to multilamellar vesicles. PRE studies with Mn^2+^ showed a higher and more uniform effect on MSI-594, contrary to a heavily shielded hydrophobic core of MSI-78. This suggests that oligomerization mainly relies on the primary sequence rather than on the membrane. Furthermore, the authors hypothesize that dimerization leads to a higher positive charge of MSI-78, increasing the interaction with anionic membranes, and to a decrease of accessible hydrophobic residues, which are buried in the dimerization interface. Another study investigated the effect of membrane lipid composition on the activity and orientation of MSI-78 [75]. It could be shown that MSI-78’s activity was inhibited by increasing amounts of anionic lipids and that it is positioned close to the surface in anionic membranes compared to a deep immersion in zwitterionic ones.

#### 4.3.2. β-Structures: Arenicin-2

**Figure 5:**
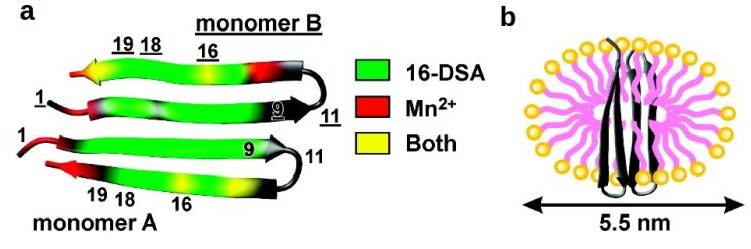
(**a**) Arenicin-2 PREs: highly affected residues by 16-DSA (green), Mn^2+^ (red) and both probes (yellow), in case of the black colored residues the PREs were below the thresholds. The proposed orientation of arenicin-2 and its interaction with a DPC micelle, including the effect of arginine 16 on the membrane’s curvature is shown in (**b**). Reproduced with permission from [77].

Arenicin-2 is a polychaeta lugworm *Arenicola marina*-derived 21-residue peptide, forming a disulfide bridge stabilized β-hairpin. In aqueous solution a right-handed twist shields the hydrophobic residues. The structure disentangles upon membrane binding, leading to an amphiphilic homodimer, where the N-terminal strands assemble in a parallel manner, exposing hydrophobic residues. [76,77] Investigation of the peptide’s backbone dynamics by NMR spectroscopy revealed fluctuations of the β-turn motif. Obviously, this peptide fluctuates between different conformations and the most populated state depends on the environment [77]. 16-DSA and Mn^2+^ ions were used to investigate the interaction of arenicin-2 with DPC-micelles [77]. Large signal reductions were found in the central parts of the β-sheet in the presence of 16-DSA, indicating a deep immersion into the micelle. In contrast, the addition of Mn^2+^ results in a relaxation enhancement for resonances belonging to the termini and the outer strands. These PREs are in agreement with a transmembrane orientation of the arenicin-2 homodimer in DPC micelles (see Figure 5).

A noteworthy detail is the high PRE for arginine 16, which is situated in the middle of the outer strands, for both probes [77]. The authors argue that it can be interpreted as the detergent head groups interacting with the positively charged side chain, thereby causing curvature of the membrane, a prerequisite for the toroidal pore mechanism. 

#### 4.3.3. Cyclotides

**Figure 6:**
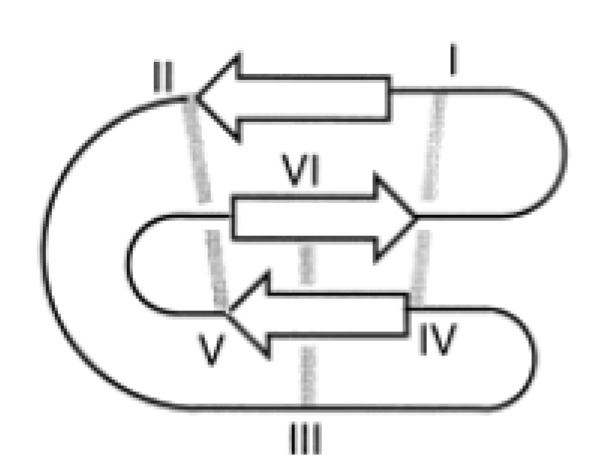
Schematic diagram of the cyclic cysteine knot with the cysteine residues labeled from I-VI and the resulting disulfide bonds shown as grey lines. Within the amide-cyclized backbone the disulfide bonds between the cysteines I and IV as well as II and V form the embedded ring being penetrated by the third disulfide bond between the cysteines III and VI. The β-sheets are represented by white arrows, forming the typical secondary structure element of cyclotides, a β-hairpin with a distorted third β-strand. Reproduced with permission from [81].

In plants, antimicrobial peptides have also been regarded for years as key players in host defense mechanisms by either providing pre-existing barriers or inducing defense responses after infection [78]. In plants up to date eight families of such peptides, being cysteine-rich and active against plant pathogens, have been identified and classified into defensins, thionins, lipid transfer proteins, snakins, MBPI and IbAMP, hevein- and knottin-like peptides containing a cysteine knot [78]. This cysteine knot [79] describes a structural motif present in these types of peptides and proteins consisting of two disulfide bonds forming an embedded ring, that is penetrated by a third disulfide bond (see Figure 6). These six conserved cysteines (I-VI) define backbone fragments of varying lengths and the sequences can be described by the formula cyclo-[CI-Xa-CII-Xb-CIII-Xc-CIV-Xd-CV-Xe-CVI-Xf] with Xa-f representing up to ten non-cysteine residues [80]. 

Cysteine knots have been discovered in many different proteins including growth factors, inhibitory proteins and toxins and based on that three classes of such disulfide-rich proteins are distinguished, namely the Growth Factor Cysteine Knots (= GFCKs), the Inhibitor Cysteine Knots (= ICKs) and the Cyclic Cysteine Knots (= CCKs), whereby this review only focuses onto the last group being represented by the plant cyclotides [81]. They are found in a broad range of plants from the *Rubiaceae* (coffee), *Violaceae* (violet) and *Cucurbitaceae* (cucurbit) family and describe the largest family of known circular proteins, so-called gene-coded miniproteins ranging in size from 20 to 40 amino acids, with a head-to-tail cyclised backbone that is stabilized by the above mentioned cysteine knot [82]. Generally, this cysteine knot is associated with a triple stranded β-sheet, as the main secondary structure element, consisting of a β-hairpin with a distorted third β-strand. The disulfide bonds stabilize the common fold by linking the β-strands that are diagonally opposite located from each other. Additionally, there is a varying number of well-ordered tight turns present in cyclotides like in other circular proteins, and in members of the Bracelet family, which is described later on, a short 3_10_ helix can be found [83]. In contrast to this, the structure of members of the Trypsin Inhibitor family, also described later, is more determined by a sequence of well-defined turns, although there is a small β-hairpin and helix turn present, too [84]. Further stabilization is achieved by hydrophobic interactions and the presence of numerous hydrogen bonds. In contrast to non-ribosomally synthesized cyclic peptides, cyclotides are translated gene products like conventional proteins except the fact that they lack an N- and C-terminus by owing an extra peptide bond linking them together [80]. Typically, proteins fold in a way to have their hydrophobic residues packed in their core. In cyclotides, however, the cysteine knot forms already a very closely packed core, which forces most of the side-chains and other hydrophobic residues to face towards the surface to avoid steric clashes resulting in the characteristic large hydrophobic patch on the surface of cyclotides. From various structural studies three subclasses of plant cyclotides have emerged, namely the Bracelet, the Moebius and the Trypsin Inhibitor cyclotides, distinguished by the fact that when describing the backbone as a ribbon, a straightforward circular ribbon depicts a bracelet, whereas a 180° twist causes a Moebius strip [81]. This twist is due to a cis-oriented peptide bond prior to a proline residue located at the tip of the β-hairpin [85,86]. 

As these plant-derived cyclotides are lacking N- and C-termini because of their circular structure, they are missing contact sites for exoproteases, as well as for endoproteases because of the restricted flexibility of their backbone. Due to the exceptional stability of their fold, resistance to thermal, enzymatic and chemical treatment and their tremendous sequence variability, while retaining the overall fold, there is an incredible potential for cyclotides in pharmaceutical applications. They show a broad range of biological activities such as, for example, potent anti-fouling activity [87], insecticidal and nematicidal [88] properties providing interesting agents for agricultural applications in the fight against plant pathogens as well as human-related bioactivities like uterotonic [89] and neurotensin inhibition [90] activities making them interesting molecular scaffolds for drug design. For example, their ability to block ion channels can be applied in pain therapy by selectively obstructing channels that are involved in pain response transmission [91]. A number of cyclotides have also been reported to possess antimicrobial [92] and antiviral activity as tested in HIV-assays [93,94], although due to their additional haemolytic activity and the lacking differentiation between protecting cells and cytotoxicity, the use as therapeutic agents is not yet possible without any further sequence modifications, although promising results have already been achieved with the cyclotide kalata B8, a hybrid of the Moebius and Bracelet family, displaying anti-HIV, but lacking haemolytic activity [95]. Cyclotides are thought to exert their biological activities through direct membrane interactions, with a stronger effect on membranes containing phosphotidylethanolamine compared to phosphocholine, rather than by specific receptors. The destabilization of the membrane caused by cyclotides can be considerably enhanced by the binding of divalent cations and either causes cell death or facilitates peptide penetration into the cell with subsequent attacking of various intracellular targets [96]. 

Kalata B1 from *Oldenlandia affinis*, a 29 amino acids comprising cyclotide, is the best studied member of the Moebius family and has been shown to be resistant to thermal as well as chemical treatment and enzymatic breakdown, conditions that normally destruct similar-sized, linear proteins [97]. It shows antimicrobial activity, likely exerted by binding to the membrane via two hydrophobic loops [96] as an initial step, which differs from the highly positively charged conventional antimicrobial peptides, followed by possible pore formation through peptide oligomers resulting in membrane rupture or peptide penetration into the cell [96]. The manner, in which the peptide interacts with the membrane, is determined by the arrangement of the hydrophobic surface patches, that are located at loop 5 and 6 in the Moebius family, which means, that all members of this family are likely to interact with the target membrane in the same mode as kalata B1, where the tip of the β-hairpin and part of the third β-strand provide the contact site [96]. 

In a study by Simonsen *et al.* [98] all non-cysteine residues were individually exchanged for an alanine to investigate the effect of each amino acid on the overall fold and the bioactivity of kalata B1. It revealed that single mutations do not affect the structure, although the bioactivity is dependent on a surface patch of mainly hydrophilic amino acids. Interestingly, these residues do not match with the hydrophobic surface patch that is required for membrane binding, leading to the assumption that the bioactivity does not rely exclusively on simple membrane interactions. Additionally, the conformation of its membrane-bound state has been determined by using the paramagnetic relaxation probes 5- and 16-doxylstearate and only slight changes in chemical shifts have been observed upon incorporation into the micelle. The overall fold of the typical triple-stranded β-sheet structure in solution, associated with the cysteine knot, is maintained in the membrane mimetic. The specific broadening of certain signals indicated that during interaction with the membrane, kalata B1 mainly remains located at the micelle surface, while the hydrophobic patch on the molecules surface gets inserted into the micelle [82]. Upon additional titration of the paramagnetic Mn^2+^, which causes cyclotides to get neutral or even slightly cationic, a relaxation enhancement could be observed, whereby the high magnitude could neither be explained by freely diffusing Mn^2+^ nor by being bound to the lipid head groups, but was rather indicative of specific binding to the cyclotide. This supports the hypothesis that binding of the divalent cation in close proximity to the hydrophobic patch helps to enhance the peptide affinity to biological membranes by increasing the amphipathicity and providing an additional contact site with the anionic lipid headgroups through the positive charge [96]. 

Kalata B7 also isolated from *O. affinis*, another member of the Moebius family with a very similar tertiary structure to the one of kalata B1, has also been studied in terms of membrane interaction and divalent cation binding [96]. By Mn^2+^ titration it has been shown, that kalata B7, like in the case of kalata B1, binds the cation at the same site and with an affinity that is comparable to the one from kalata B1. The interaction of the peptide with DPC micelles was investigated by using NMR diffusion measurements. The paramagnetic 5-doxylstearate was used to determine the orientation of the peptide with respect to the micelle surface, which showed that two parts of the peptide, namely the hydrophobic regions of loop 2 and 5 insert into the interior of the detergent micelle. Like with kalata B1 the overall fold is maintained upon binding to the membrane mimetic. In spite of the similar structure both peptides show significantly different allocation of their hydrophobic surface regions. The substitution of an amino acid in the region of the β-hairpin in kalata B1 with a positively charged residue in kalata B7 introduces a gap in the large hydrophobic surface patch, whereas a dipeptide replacement in loop 2 increases the hydrophobicity in kalata B7 [96]. The resulting smaller interaction interface for kalata B7 compared to kalata B1 leads to different ways of interaction and differently oriented peptides with respect to the micelle surface as mentioned above.

Cycloviolacin O2 from *Viola odorata*, a representative of the Bracelet family, has been described to have strong cytotoxic activity due to its ability to disrupt membranes [99] and has also been studied in terms of membrane interaction [100]. The orientation of the peptide bound to DPC micelles has been determined again by using the paramagnetic 5- and 16-doxylstearate as with kalata B1. Like it is the case with members of the Moebius family, the predominant secondary structure element is a β-hairpin, whereby members of the Bracelet family additionally harbour a short α-helix that is missing in the Moebius family. Moreover, the hydrophobic surface patches are located differently in both cyclotide subclasses. Whereas it stretches over loop 2 and 3 in Cycloviolacin O2, it comprises loop 2 and 5 in kalata B1 and B7 respectively. [86,96,100] Similar to members of the Moebius family, the structures of representatives from the Bracelet family are also stabilized by a network of several backbone hydrogen bonds. Cycloviolacin O2 shows little structural change in the membrane-bound form compared to the structure of the free cyclotide and it was demonstrated to bind close to the surface of the DPC micelle with the loops containing the hydrophobic regions showing the largest shift changes upon binding. This was further indicative of an altered orientation in the membrane mimetic, which was determined by the above mentioned paramagnetic probes. By monitoring the signal broadening with a series of NOESY spectra, it could be observed that 5-doxylstearate leads to more signal attenuation than 16-doxylstearate, suggesting a binding close to the micelle surface [100]. 

With these studies on three different cyclotides from the two major subclasses it could be shown, that the global fold is conserved throughout the different families and that differences in the membrane binding mode and the orientation relative to the micelle surface are determined by differences in the presence of charged residues and the arrangement of hydrophobic surface patches.

#### 4.3.4. AMPs and MD Simulations

Due to the fact that membrane association and tertiary structure formation/rearrangements happen on a sub-millisecond timescale [101], they are difficult to study by conventional techniques. Here, molecular dynamics (MD) simulations can provide a major contribution [102,103]. RP-1 is an AMP based on the antimicrobial domain of the platelet factor-4 class proteins [102]. A combination of NMR and MD simulation was applied not only to solve the structure but also to get insight into peptide-lipid interactions [102]. RP-1 adopts an amphipathic α-helical conformation with only minimal differences in side-chain arrangements in SDS compared to DPC micelles. Highest differences are observed for lysine 8 and 9, which can probably be explained by different electrostatic interactions with the detergents. Penetration into the micelles was investigated by the incorporation of 16-DSA. The measurements showed the typical PRE profile for amphiphilic peptides in a parallel orientation to the membrane, and a slightly deeper insertion of the N-terminal parts compared to the C-terminal ones. Unfortunately, a comparison between the two different types of micelles cannot be made, as one cannot rule out that 16-DSA is differently embedded in SDS and DPC respectively. Considering these results, it seems that the main differences in the interaction with a certain membrane are sidechain lipid specific interactions. MD simulations were carried out to address this issue. Whereas in DPC micelles RP-1 stayed rather close to the surface without further impact on the micelles’ shape, in SDS micelles the peptide association led to a toroidal structure, which coincides with a deeper penetration of the peptide into the hydrophobic core.

Another example is the study of Dittmer *et al.* investigating the binding of alamethicin from the fungus *Trichoderma viride* to 1,2-dihexanoyl-sn-glycero-3-phosphocholine/1,2-dimyristoyl-sn-glycero-3-phosphocholine (DMPC/DHPC) bicelles [104]. This is highly challenging for solution NMR, not only because of the size of the complex, but also because of the presence of high amounts of the rare aminoisobutyric acid, which lacks α-protons. Interestingly, the line width of the signals is quite narrow considering the size of the complex (453 kDa), which points to high dynamics of the peptides embedded within the membrane. Alamethicin adopts a helical structure with a kink caused by the G-x-x-P motif. Lipid peptide NOEs as well as PRE measurements with Gd(DTPA-BMA) imply a transmembrane orientation. However, the high PRE for the glutamine 7 sidechain could not be easily explained, since it should be buried in the bicelle’s interior. A potential pore formation was precluded, as the addition of Dy^3+^, which in contrast to Gd(DTPA-BMA) would be able to diffuse into the pore, resulted in similar PREs. MD simulations reveal a preferred Gln7 sidechain conformation, which is close to the bicelle’s lipid head groups and the proximity to the solvent can lead to the transfer of the water’s relaxation enhancement.

### 4.4. Amyloid Peptides

**Figure 7:**
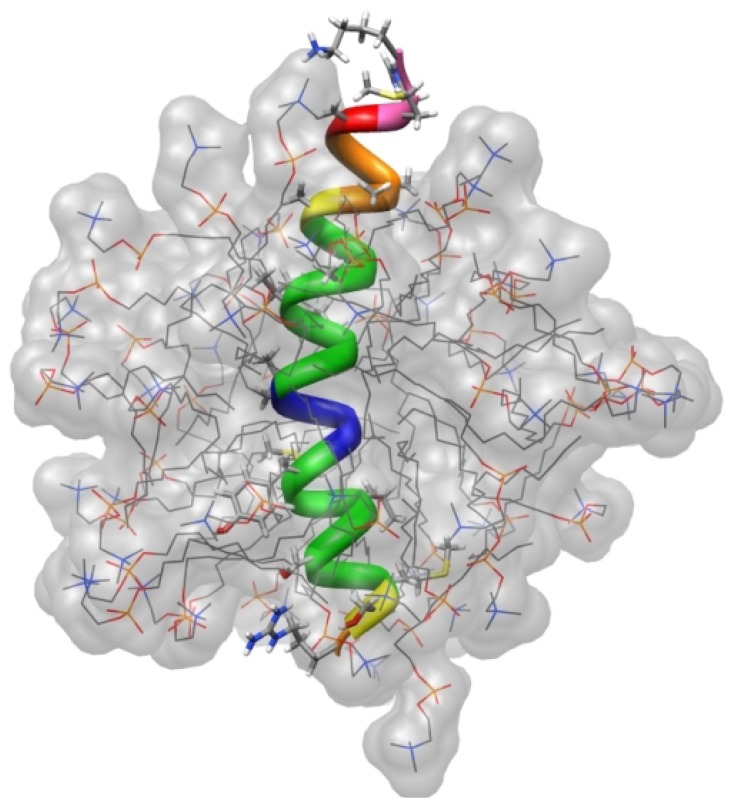
Position of PrP(110–136) in modeled DPC micelles. PREs induced by Gd(DTPA-BMA) are color-coded from low (blue) to high (red-pink). Reproduced with permission from [105].

Deposits of amyloid peptides and proteins such as amyloid β-protein (Aβ), islet amyloid polypeptide (IAPP or amylin) and semen-derived enhancer of viral infection (SEVI) are involved in a variety of human diseases, including Alzheimer, Parkinson, Huntington and Creutzfeld-Jakob diseases, type II diabetes or HIV infection. They often contain hydrophobic regions, which bind to membranes. As an example, Aubin *et al.* [105] determined the structure and orientation of the conserved hydrophobic region (residues 110–136) of the human prion protein PrP(110–136) in DPC micelles. This peptide forms one stable α-helix in membrane-mimetics. The orientation was obtained by measuring signal intensity changes in ^1^H-^15^N-HSQC spectra in the absence and presence of Gd(DTPA-BMA). PrP(110–136) inserts into DPC micelles in a nonsymmetrical fashion. The N-terminus shows higher solvent PREs than the C-terminus, which indicates that the N-terminus is closer to the surface or even sticks out of the micelle. This localization of the peptide allows interactions between the guanidine group of Arg136 and the polar headgroups of DPC and also between the most hydrophobic region of PrP(110–136) with the core of the micelle (see Figure 7).

For each of these polypeptides that are disordered in solution, the neurotoxic state is attained through self-assembly into stable amyloid fibrils via a conformational transition from an α-helical structure upon membrane-binding to the final common β-strands association [106]. The 40 residue spanning peptide Aβ(1–40), which is related to the Alzheimer’s Disease, is the major amyloid plaque forming component after proteolytic cleavage from amyloid precursor proteins (APPs) and comprises a central hydrophobic cluster being responsible for the peptide aggregation upon membrane interaction [107,108]. PRE studies with Mn^2+^ and 5-doxyl stearic acid were carried out to study the positioning of the peptide in SDS micelles. It was shown that interaction with the membrane induces the formation of two helices in the centre and close to the C-terminus respectively, with their signal intensities being most affected by 5-doxyl stearic acid, while on the contrary those parts are well-protected from exposure to the Mn^2+^ ions, which clearly affect the unstructured termini as well as the central region between the helices [107]. The obtained data from this study suggest that the helices are located inside the micelle, with the central helix close to the surface and the C-terminal one buried into the interior of the micelle, while the flexible parts are located outside or at the surface of the membrane mimetic. Another study was carried out by Yamaguchi *et al.* to investigate the interaction between the two most common isoforms of Aβ, the above mentioned Aβ(1–40) and the more fibrillogenic and neurotoxic Aβ(1–42) by using PRE studies with the nitroxide radical MTSL [109].

An α-helix formation has also been found for the 37 residue peptide hormone islet amyloid polypeptide (IAPP or amylin) in DPC [110] and SDS micelles [111]. IAPP is involved in glucose metabolism by interplaying with insulin and is found in amyloid deposits in the pancreas of type II diabetes patients. The structure of rat IAPP, which does not aggregate as easily as the human form and is less toxic, shows an α-helix between residues 5–23 and a disordered C-terminal region. Based on solvent PREs in the presence of Mn^2+^ the peptide is bound at the surface of the membrane-mimetic and the C-terminal region is freely mobile in solution [110]. The solution NMR structure of the human IAPP in SDS micelles has shown a hairpin loop for the N-terminal four residues due to a single disulfide bond between the cysteines at position 2 and 7, with residue 7 already belonging to the α-helix, which spans amino acid 5 to 28 and forms the core of the structure. Paramagnetic quenching studies with Mn^2+^ and 16-doxyl-stearate proved that the human IAPP binds parallel to the surface of the membrane mimetic with the segment from 5–20 immersed and the C-terminal part positioned at the solvent interface [111]. Another example of amyloid peptides is the 39 residue fragment of the prostatic acid phosphatase, PAP_248-286_, which has been found to enhance HIV infection via the formation of amyloid fibers called SEVI [112]. In contrast to the helical conformation formed in most amyloid peptides upon membrane binding, PAP_248-286_ remains mainly disordered with only comprising an α-helical centre spanning residues 262 to 270 apart from the flexible termini, although it undergoes a conformation change to the characteristic β-sheet upon aggregation, too. PRE measurements show nearly complete quenching of all signal intensities, indicating that the peptide is more solvent exposed compared to other amyloidogenic peptides.

### 4.5. Cell Penetrating Peptides

Hydrophilic macromolecules usually enter cells via a process called endocytosis by initial absorption to the membrane-bound receptor or directly to the membrane, prior to an energy-dependent vesicle formation. These molecules are then addressed to various cellular compartments or released into the cytoplasm [113]. In contrast to this process, penetration is apparently an energy- and receptor-independent, and therefore cell-unspecific way of translocating peptides across the membrane, additionally allowing conjugated cargoes to be targeted into the cytoplasm or the nucleus of any type of cell [114]. Such cell-penetrating peptides have shown the ability to transport macromolecules of sizes that exceed their own molecular weights up to several times making them attractive for therapeutical applications [114]. These peptides are classified into protein-derived and synthetic and/or chimeric cell-penetrating peptides, whereby the previous ones are further divided into homeodomain-derived, Tat-derived and signal-sequence based peptides [114]. Homeodomains define protein segments comprising approximately 60 amino acids, through which homeoproteins, a class of transcription factors, can bind DNA and cross cell membranes via secretion or internalization. Tat-derived peptides are involved in HIV replication and signal-sequence based peptides play a role in addressing premature proteins to the membranes of target intracellular compartments [114], whereby we focus here on members of the homeodomain-derived and synthetic cell-penetrating peptides.

One member of the homeodomain-derived peptides is penetratin, a 16 residue peptide from the *Drosophila antennapedia* transcription factor, which has the same ability of penetrating cell membranes while carrying macromolecules like the full length DNA-binding homeodomain [115]. Structural studies with SDS micelles [116] and negatively charged phospholipid vesicles [117] showed that upon interaction with the membrane mimetics penetratin adopts an α-helical conformation, whereas the N- and C-terminal part remain unstructured. Paramagnetic probes such as Mn^2+^, 5- and 12-doxyl stearic acid were applied to determine the orientation of penetratin in the micelle, with expected broadening effects from amino acids outside the SDS micelle, close to the surface or deep immersed [115]. The combined data showed that penetratin has its N-terminal part close to the surface and the C-terminal part placed in the micelle center [118]. Additionally, the structure and orientation of this peptide in DMPG/DMPC bicelles has been investigated, [119]. Whereas penetratin has a mainly random coil structure in solution, it adopts an α-helical conformation in bicelles, as was already observed with micelles and vesicles. [116,117] The orientation of the peptide in the bicelle was determined with the same paramagnetic probes as mentioned above and it could be observed that penetratin locates more or less parallel at the interface of the hydrophobic interior and the phospholipid head groups [119]. 

A previous study [120] described a model, in which hydrophilic molecules associated to cell penetrating peptides, such as penetratin, can be translocated between cells. In this model, binding of penetratin to negatively charged phospho- or glycolipids respectively on the outer face of the membrane results in a membrane destabilization with subsequent change from a phospholipid bilayer to an inverted micelle, which then allows the accommodation of the peptide in its hydrophilic interior and by this a translocation of peptides and their cargoes up to a certain size across the plasma membrane.

Galparan describes a chimeric peptide with the N-terminal part taken from the neuropeptide galanin, which is participating in pain signaling, and the C-terminal part from the wasp venom mastoparan, which is acting as a mast cell degranulating peptide [114]. Structural studies of galanin bound to SDS micelles have been carried out previously [121] and its orientation in the membrane mimetic was determined by using the same paramagnetic probes as mentioned above for penetratin [115]. Galanin has a rather random conformation with three well-defined turn regions and resides close to the surface of the SDS micelle with two regions comprising four to six residues and two single amino acids sticking out into the solvent [121]. By substituting a proline residue in the middle of the amino acid sequence of galparan with a lysine for convenient association of cargo molecules to its side chain, a new chimeric cell-penetrating peptide, the 27 residue comprising transportan, was created and its binding properties to and positioning in SDS micelles were investigated with Mn^2+^ and spin-labeled 5- and 12-doxylstearate respectively [115]. The study also comprised investigation of the properties of mastoparan alone, to allow comparison between the naturally occurring peptides galanin and mastoparan and the synthesized chimeric peptide transportan. It has been shown that the C-terminal mastoparan part adopts an α-helical conformation and most of it is immersed into the micelle interior with only the outermost C-terminal amino acids surface exposed, whereas the N-terminal galanin part resides close to the surface of the SDS micelle. This demonstrates that both parts of the chimeric peptide show the same properties as the galanin and mastoparan peptides [115]. 

Additional studies of transportan in neutral phospholipid bicelles [122] showed the same results as with SDS micelles, except that for the galanin part also a tendency to adopt an α-helical conformation could be observed, stating that at least in the case of this chimeric peptide the two different membrane mimetics are nearly equivalent for studying the structure and orientation.

## 5. Larger Membrane-Mimetic Systems

The majority of solution NMR studies on membrane-bound peptides and proteins has been carried out on micelles due to their relatively fast tumbling and resulting in better resolved NMR signals [8,9]. However, these small micelles (diameter ~4 nm) are characterized by a high surface curvature. To get information about the topology of membrane-bound biomolecules in more physiological membrane-mimetics an increasing number of reports describe the use of bicelles and small unilamellar vesicles. Intriguingly, the very first papers on the use of paramagnetic effects on NMR signals for obtaining topological information of membrane-bound molecules were obtained with vesicles [123]. Information about the localization of tryptophan residues of gramicidin A’ in phosphatidylcholine vesicles was obtained by the addition of paramagnetic lanthanides (Tm^3+^, Eu^3+^, Dy^3+^ and Pr^3+^) [124]. Due to partial binding of these ions to the surface of the vesicles, changes in the chemical shifts of tryptophan signals have been observed, which indicated that these residues are binding to the phosphate regions. A more quantitative evaluation of PREs induced by Mn^2+^ and Tm^3+^ ions and nitroxide labeled stearic acid attached to the phosphatidylcholine vesicles later lead to the conclusion that in gramicidin A dimers the N-terminal end is inside the membrane-mimetic and the C-terminus close to the surface [125]. For these earlier studies of peptides in vesicles one-dimensional NMR spectra were acquired. Due to the limited spectral resolution only a few PREs or lanthanide induced shift changes could be observed and the investigated peptides were partially labeled with ^13^C and ^19^F to obtain site-specific information. In addition, vesicles are very large for solution NMR studies and the line-widths of NMR signals are extensively broadened. More recently isotropic bicelles were introduced in the field of solution NMR [126] and besides their widespread use as alignment media for measuring residual dipolar couplings [127], they also provide a membrane-mimetic much closer to biological membranes than micelles. Using isotropic bicelles consisting of dimyristoylphosphatidylcholine (DMPC) and dihexanoyl-phosphatidylcholine (DHPC) the structure and orientation of the ionophore polyether antibiotic salinomycin could be obtained. PREs were measured as actual relaxation rate enhancements by inversion recovery experiments in the absence and presence of Mn^2+^ and 5- and 12-doxylstearic acid. It was found that this peptide is immersed shallowly in the flat DMPC rich region of the bicelles [128]. Mixed DMPC/DHPC bicelles were also used in another, highly interesting, report on the structure, dynamics and orientation of the small multidrug resistance transporter EmrE. Henzler-Wildman *et al.* [129] found this protein to form asymmetric dimers, where both monomers have opposite orientations in the membrane-mimetic. Using gadobenate dimeglumine as a paramagnetic agent different sets of PREs were found for both monomers using TROSY-type spectra. These results confirmed the model of exchange between an inward and outward facing dimer of EmrE. The protein is open to one side of the membrane and conformationally interconverts upon substrate binding. 

## 6. Conclusion and Outlook

In the last ~35 years a large number of solution NMR studies using paramagnetic additives on membrane-binding peptides and proteins appeared and many questions, in particular about the structure and orientation in the membrane could be answered. The systems studied and reviewed here include many antimicrobial peptides, toxins and peptides derived from large membrane-bound proteins. While the atomic resolution structures and topology in the membrane of these small peptides can be investigated by routine NMR methods the inherent size limitation of solution NMR has kept the number of reports on membrane-bound proteins beyond 5 kDa still very small. Another field which has just started to be investigated is the use of larger membrane-mimetic systems and even whole, living cells for NMR studies. Further research in this direction would be fruitful to prevent any artificial effects resulting from the large surface curvature of micelles, which are currently mainly used. 
